# Transcriptional states and chromatin accessibility during bovine myoblasts proliferation and myogenic differentiation

**DOI:** 10.1111/cpr.13219

**Published:** 2022-04-01

**Authors:** Qian Li, Yahui Wang, Xin Hu, Yapeng Zhang, Hongwei Li, Qi Zhang, Wentao Cai, Zezhao Wang, Bo Zhu, Lingyang Xu, Xue Gao, Yan Chen, Huijiang Gao, Junya Li, Lupei Zhang

**Affiliations:** ^1^ Institute of Animal Sciences, Chinese Academy of Agricultural Sciences, Key Laboratory of Animal (Poultry) Genetics Breeding and Reproduction Ministry of Agriculture and Rural Affairs Beijing China

## Abstract

**Objectives:**

Although major advances have been made in bovine epigenome study, the epigenetic basis for fetal skeletal muscle development still remains poorly understood. The aim is to recapitulated the time course of fetal skeletal muscle development *in vitro*, and explore the dynamic changes of chromatin accessibility and gene expression during bovine myoblasts proliferation and differentiation.

**Methods:**

PDGFR‐ cells were isolated from bovine fetal skeletal muscle, then cultured and induced myogenic differentiation *in vitro* in a time‐course study (P, D0, D2,and D4). The assay for transposase‐accessible chromatin sequencing (ATAC‐seq) and RNA sequencing (RNA‐seq) were performed.

**Results:**

Among the enriched transcriptional factors with high variability, we determined the effects of *MAFF*, *ZNF384*, and *KLF6* in myogenesis using RNA interference (RNAi). In addition, we identified both stage‐specific genes and chromatin accessibility regions to reveal the sequential order of gene expression, transcriptional regulatory, and signal pathways involved in bovine skeletal muscle development. Further investigation integrating chromatin accessibility and transcriptome data was conducted to explore cis‐regulatory regions in line with gene expression. Moreover, we combined bovine GWAS results of growth traits with regulatory regions defined by chromatin accessibility, providing a suggestive means for a more precise annotation of genetic variants of bovine growth traits.

**Conclusion:**

Overall, these findings provide valuable information for understanding the stepwise regulatory mechanisms in skeletal muscle development and conducting beef cattle genetic improvement programs.

## Graphical abstract

Here, we conducted ATAC‐seq and RNA‐seq to profile chromatin accessibility and gene expression dynamics over a time course spanning from bovine foetal myoblasts proliferation and myogenic differentiation. Additionally, we integrated our data with results from genome‐wide association study (GWAS) of bovine growth traits to characterize the functions of these regulatory elements. Taken together, our study demonstrated a step‐wise dissection of the transcriptional regulation network for skeletal muscle development and provide a systematic understanding of the molecular circuits governing skeletal muscle development.

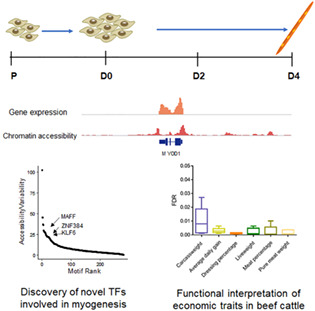

## 1 INTRODUCTION

Skeletal muscle, which accounts for about 40% of total body weight,^1^ is a highly dynamic tissue of the body. The growth and maintenance of skeletal muscle play an important role in crucial physical and metabolic function in human, while in animal production, affecting growth efficiency and meat quality.^2^ The growth of skeletal muscle fibres is initiated during embryonic stage,^3^ then is fixed during the foetal stage, and remains almost unchanged in number after birth.^4^ Notably, the foetal stage is the critical period of myogenesis, when the majority of skeletal muscle fibres form.^5^ It has become established that many specific signalling pathways and transcription factors (TFs) are involved in myogenesis.^5^, ^6^, ^7^, ^8^ Wingless and Int (Wnt), paired box 3 (*Pax3*), and *Pax7* are necessary myogenic regulatory factors (*MRFs*) that regulate the myogenic lineage differentiation of mesenchymal stem cells during embryo stage.^5^, ^9^, ^10^, ^11^, ^12^ Although previous studies provide strong evidence of the contribution by *MRFs* to skeletal muscle development,^13^, ^14^ the precise mechanisms underlying skeletal muscle development remain unclear. Halstead et al. reported the dynamic changes of chromatin accessibility during bovine pre‐implantation development, indicating their important roles in species‐specific embryonic genome activation.^15^ In addition, a comprehensive functional annotation of bovine rumen epithelial cells has been well established and provided important information to explore butyrate‐induced biological effects in bovine.^16^ As Functional Annotation of Animal Genomes (FAANG) Consortium proposed, additional efforts have been made to investigate chromatin accessibility related to skeletal muscle development.^17^, ^18^, ^19^ However, particularly in bovine models, previous studies in the context of skeletal muscle chromatin accessibility have mainly focused on comparative analysis across distant species to identify tissue‐specific regulatory elements.^20^, ^21^, ^22^ Recently, Cao et al. described comparative analysis of enhancers between adult and embryo bovine muscle using chromatin accessibility.^23^ Despite the foetal stage is critical for skeletal muscle development, only limited evidence of foetal muscle growth and development was available from prior studies, and these findings may ignore that skeletal muscle development is a dynamic process comprised of multiple developmental stages. In our study, we reconstructed the course of myogenic differentiation in vitro, providing an ideal model to define when and how these regulatory changes occur during skeletal muscle development.

## 2 MATERIALS AND METHODS

### 2.1 Primary cells isolation and culture

The myoblasts were enzymatically isolated from longissimus dorsi tissues of bovine foetuses at 90 days, and cultured in low‐glucose DMEM with 10% foetal bovine serum (growth medium) as described previously.^24^ At 90% confluence, the cells were trypsinized with 0.25% trypsin–EDTA (Gibco, Grand Island, NY) and passaged to culture plates. At 100% confluence, the growth medium was exchanged by low‐glucose DMEM with 5% horse serum (differentiation medium). The differentiation medium was then changed every 2 days.

### 2.2 RNA extraction, reverse transcription and quantitative real‐time PCR

Total RNA was isolated using the TRIzol reagent (Invitrogen Life Technologies). RNA concentration and quality were determined by NanoPhotometer N50 (Implen, Munich, Germany). Next, the qRT‐PCR was performed using ABI QuantStudio 7 Flex system (Life, Carlsbad, CA) in accordance with the instructions of KAPA SYBR® FAST qPCR Kit (KAPABiosystems, Wilmington, MA). The primer sequences involved in qRT‐PCR were listed in Table S1.

### 2.3 siRNA transfection

Small interfering RNA (siRNA) were supplied by RiboBio (Guangzhou, China), and the sequences are listed in Table S2. With the help of LipofectamineTM RNAiMAX (Invitrogen Life Technologies, Carlsbad, CA) reagent, the siRNAs against *MAFF*, *ZNF384*, *KLF6* (*siMAFF*, *siZNF384*, *siKLF6*) or siRNA control (NC) were transfected into myoblasts at a final concentration of 50 nM.

### 2.4 CCK8 assay

After interference of *MAFF* in myoblasts, CCK8 reagent was added at 12, 24, 36, 48 and 60 h. After continuously incubating for 2 h, the absorbance (OD values) at 450 nm was then measured.

### 2.5 mRNA‐seq library construction, sequencing and data processing

mRNAs were purified from total RNAs using oligo‐dT beads and then fragmented with Mg^2+^. RNA‐seq libraries were constructed by following steps: reverse transcription, the end repair of cDNA, poly(A) tail and index addition. All libraries were sequenced on the HiSeq2500 platform following a PE150 strategy. RNA‐seq datasets from each sequencing library were trimmed with Trim_Galore 0.6.3 to remove Illumina adapter sequences and low‐quality sequences. Trimmed reads were then aligned to bovine genome (ARS‐UCD1.2) using STAR 2.7.3a.^25^ Gene expression counts were calculated by the ‐‐quantMode GeneCounts functionality in STAR. For downstream analysis, RNA‐seq raw counts were normalized by computing counts per million and then formed to Z scores. Differential gene expression analysis was performed by DESeq2 (adjusted *p*‐value <0.01).^26^, ^27^

### 2.6 ATAC‐seq library construction, sequencing and data processing

The ATAC‐seq libraries were performed as TruePrep DNA Library Prep Kit V2 for Illumina (Vazyme, Nanjing, China). Briefly, 5 × 10^4^ cells were counted and resuspended in precooled lysis buffer. The unfixed nucleuses were incubated with Tn5 transposase at 37°C for 30 min. Amplified the fragments for 16 cycles and purified using the VAHTS RNA Clean Beads (Vazyme, Nanjing, China). Libraries were quantitated using Qubit 4 Fluorometer (Invitrogen, Singapore), and then sequenced on Illumina HiSeq2500 platform following a PE150 strategy. ATAC‐seq datasets from each sequencing library were first trimmed with Trim Galore 0.6.3 to remove Illumina adapter sequences and low‐quality sequences. Trimmed reads were then aligned to bovine genome (ARS‐UCD1.2) using Bowtie2 2.3.5.^28^ ATAC‐seq peak calling for each individual replicate was performed by MACS2 2.1.2.^29^ To establish a common peak set, peak summits were extended ±250 bps to obtain 500 bp fixed‐width peaks.^30^ Overlapping peaks in each sample were handled using an iterative removal procedure with the strongest signal (−log_10_ FDR) until all peaks were accounted. FeatureCounts^31^ was used to quantify the raw read counts from the common peak set including all replicates. For downstream analysis, ATAC‐seq raw counts were normalized by computing counts per million and then formed to *Z* scores. Differential chromatin accessibility analysis was performed by DESeq2 (adjusted *p*‐value < 0.01 and |log2FC| > 2). Peak annotation was performed by R package ChIPseeker.^32^

### 2.7 Clustering and visualization of ATAC‐seq and RNA‐seq signal

For clustering, the normalized counts of all genes and the top 50,000 open chromatin peaks (ranked by adjusted *p*‐value) were used for principal component analysis (PCA) and Spearman correlation analysis using prcomp_irlba and Complexheatmap.^33^ The coverage tracks were generated by deeptools 3.3.0 bamCoverage.^34^ Visualization of ATAC‐seq and RNA‐seq coverage track was performed by IGV 2.6.2.^35^ Each read was extended by 250 bp and the genome‐wide signal was generated by the normalized read counts (CPM) per bin. TF deviation and variability analysis were performed via the motifmatchr package, and CisBP transcription factors from the ‘human_pwms_v2’ dataset.^36^ Transcription factor activity scoring was performed using the 501‐bp fixed‐width peak set, which combined replicates for each differentiated time point.

### 2.8 Identification of stage‐specific genes and stage‐specific peaks

We used a Shannon‐entropy‐based to identify differentiation stage‐specific genes/peaks as previously described.^37^, ^38^ We first selected those with entropy scores less than a predefined threshold (stage‐specific genes: 1.6; stage‐specific peaks: 1.8) as candidates. Then the ATAC‐seq peak was highest active in this stage (normalized CPM > 1), and its high activity (normalized CPM > 0) could not be observed at more than two additional stages. The enrichment of known motifs for stage‐specific peaks was detected by findMotifsGenome.pl function of the HOMER package.^39^ After filtering out motifs of TFs which were not expressed in all stages (MAX_(CPM)_ < 1), the top enriched known motifs were reported.

### 2.9 Functional annotation enrichment analysis

Gene Ontology (GO) enrichment analysis for the tested genes was performed by DAVID V 6.8.^40^, ^41^ KEGG pathway enrichment was conducted by KOBAS 3.0.^42^

### 2.10 Integration of ATAC‐seq and RNA‐seq

Based on genomic region, consensus peaks were classified into six groups: Promoter (TSS ±2.5 kb), 5′UTR, 3′UTR, downstream, exon, intergenic and intron. Spearman's correlation coefficients were calculated between gene expression levels (normalized CPM) and chromatin accessibility levels (normalized CPM) of each genomic region group. We further defined genes/peaks based on the previous definition^43^: Genes defined as HA/HE that maximum value of ATAC‐seq/RNA‐seq reads was higher than the 70th percentile; Genes/peaks were defined as MA/ME that maximum value of ATAC‐seq/RNA‐seq reads was below the 50th percentile. Each gene associated with promoter‐region accessibility was assigned to four groups: HA–HE, high accessibility/high expression; MA–ME, medium‐low accessibility/medium‐low expression; HA–ME, high accessibility/medium‐low expression; and MA–HE, medium‐low accessibility/high expression.

### 2.11 Enrichment analysis based on empirical sampling

Overall, 3804 human housekeeping genes were converted successfully to 3256 bovine orthologous housekeeping genes using the BioMart tool.^44^, ^45^ Enrichment analysis was conducted on housekeeping genes enriched in HA–MA group and gene set randomly sampled 10,000 times. The empirical *p*‐value was calculated as:

$$p-\text{value}\frac{1+\sum_{i=1}^{10,000}\;\left(\text{number of}\;\left({\text{proportion}}_{\left(\text{random sample}\right)}>{\text{proportion}}_{\left(\mathrm{HA}-\mathrm{MA}\;\text{group}\right)}\right)\right)}{10,001}.$$

QTL regions and associated SNP that affect cattle anatomy and growth were derived from Animal QTL Database (Cattle QTLdb: https://www.animalgenome.org/cgi-bin/QTLdb/BT/index).

### 2.12 Genome‐wide association analyses and GWAS enrichment analysis

A total of 1233 Chinese Simmental beef cattle were derived from Ulgai, Xilingol League, and Inner Mongolia, China from 2008 to 2015 as previously described.^46^, ^47^ Phenotypic data including carcass weight, average daily gain, liveweight, dressing percentage, meat percentage and pure meat weight were collected. DNA samples were genotyped on Illumina BovineHD 770 K SNP array, and then imputed to whole‐genome sequence level.^47^ Genome‐Wide Association Analyses were performed by GCTA version 1.93.0.^48^, ^49^ The annotation of GWAS Summary Statistics was lifted over from UMD 3.1 to ARS‐UCD1.2 via the UCSC liftOver tool.^50^ To check whether the SNP effects were more enriched in chromatin peaks than background regions, GWAS signals enrichment analysis were performed using Perl scripts (sumGSE, https://github.com/WentaoCai/GWAS_enrichment).^51^

## 3 RESULTS

### 3.1 Comprehensive profiling of the chromatin accessibility and transcriptional landscape

**FIGURE 1:**
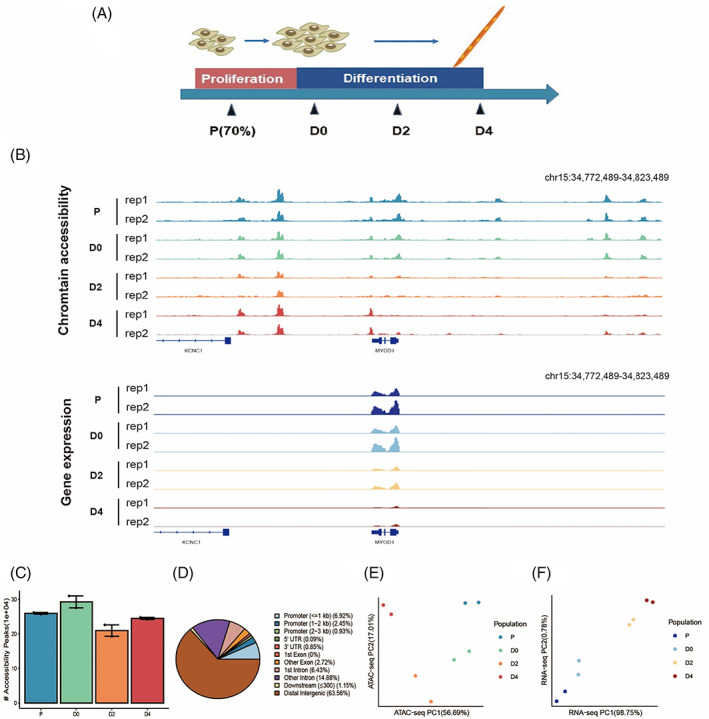
Comprehensive profiling of the chromatin accessibility and transcriptional landscape during bovine myoblast proliferation and myogenic differentiation. (A) Schematic illustration of myogenic differentiation *in vitro*. P, proliferation; D0, D2, D4, myogenic differentiation after 0, 2 and 4 days. (B) Normalized coverage (CPM) of chromatin accessibility and mRNA levels around *MYOD* gene for each time point. (C) Number of chromatin accessibility regions detected at P, D0, D2 and D4 time points. (D) Genomic distribution of chromatin accessibility peaks from the common peak set, namely promoter (TSS ± 2.5 kb), 5′UTR, 3′UTR, downstream, exon, intergenic and intron. (E) and (F) Principal component analysis (PCA) plot of chromatin accessibility and gene expression during myogenesis in two dimensions (PCA1 and PCA2). The normalized counts of all genes and the top 50,000 open chromatin peaks (ranked by −log_10_ FDR) were used for plot

To investigate the molecular control during bovine myoblast proliferation and myogenic differentiation, we isolated PDGFR^−^ cells from bovine foetal skeletal muscle, then cultured and induced myogenic differentiation *in vitro* in a time‐course study. Next, we profiled the transcriptome and chromatin accessibility during bovine myogenesis at four important time points, including proliferation (P) and myogenic differentiation after 0, 2, and 4 days (D0, D2, and D4) (Figure 1A,B). Overall, we generated an average of 61.23 million uniquely mapped reads for ATAC‐seq library and an average of 21.55 million mapped reads for RNA‐seq library (Tables S3 and S4). Fragment size distribution displayed a clear nucleosomal periodicity in ATAC‐seq and the expression pattern of major myogenic regulatory factors in RNA‐seq demonstrated the robustness of our dataset (Figure S1A,B). To enable direct comparison of peaks across different time points during bovine myogenesis, peaks called from all replicates (range 192,928 to 309,729) were pooled together and identified regions of open chromatin, resulting in a 501‐bp fixed‐width peak set with 379,553 accessible chromatin peaks (Figure 1C). To predict genomic features of functional regulatory elements, annotation of accessible regions was performed (Figure 1D). As expected, the majority of consensus peaks identified in the peak set were assigned to distal intergenic (63.56%), intronic (21.21%) and promoter (10.30%) regions throughout the genome, consistent with previously reported profiles of chromatin accessibility in cattle.^20^ We also performed the Spearman correlation and principal component analysis on both gene expression and chromatin accessibility profiles, and our data suggested a continuous trajectory of bovine myogenesis and a high correlation between biological replicates (Figures 1E,F and S2A,B). Thus, examples of the chromatin accessibility and transcriptomic dynamic changes during bovine myoblast proliferation and myogenic differentiation at myogenic marker genes were shown to demonstrate the robustness of our dataset (Figures 1B and S2C).

### 3.2 Time‐dependent modules of transcriptional regulation during bovine skeletal muscle development

**FIGURE 2:**
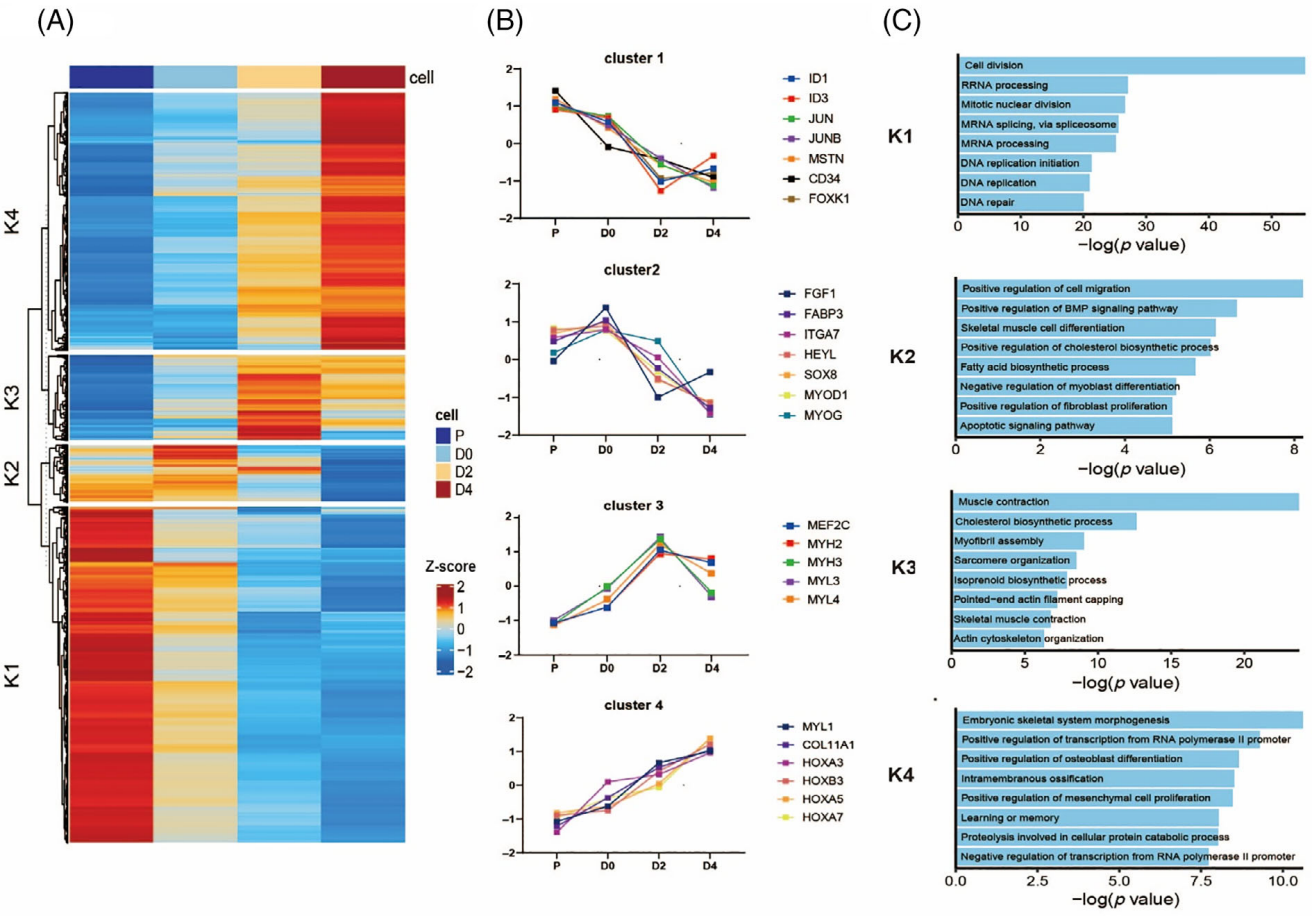
Genome‐wide transcription profiles of differentially expressed genes (DEGs) from time‐point pairwise comparisons. (A) Heatmap of differentially expressed genes from time‐point pairwise comparisons, yielding four clusters of co‐regulated gene sets. (B) Examples of co‐regulated gene sets showed similar gene expression pattern in each cluster. (C) Enrichment gene ontology (GO) terms of co‐regulated gene sets in each cluster. Only GO terms for GOTERM_BP_DIRECT and *p*‐value <0.05 were considered

To characterize transcriptional modules during bovine skeletal muscle development, we defined gene expression profiles at four differentiated time points and identified a total of 4655 differentially expressed genes (DEGs) among each pairwise comparison. We also employed k‐means clustering to separate differentially expressed genes, yielding four clusters of co‐regulated gene sets that showed a wide range of expression patterns (Figure 2A,B). For example, many up‐regulated genes in the early stages of myoblasts proliferation were related to cell proliferation and cell cycle progressions such as *ID1*, *ID3*, *JUN*, *JUNB* and *MSTN*.^52^, ^53^ *HOX* genes *(HOXA3*, *HOXB3*, *HOXA5*, *HOXA7*) involved in embryonic skeletal system morphogenesis were strongly up‐regulated in the later stages of myogenic differentiation.^54^ Likewise, the marker genes of myogenic myogenesis including *MYOD*, *MYOG* showed maximum expression activity in the early stages, while *MYHs* (*MYH2*, *MYH3*), *MYLs* (*MYL3*, *MYL4*) in the later stages. We further performed Gene Ontology (GO) analysis on the differentially expressed genes within each cluster and identified associated GO terms to gain insights into variable changes of gene expression during the bovine skeletal muscle development (Figure 2C). The genes in cluster K1 demonstrated a highly significant enrichment for cell division‐related processes, suggesting their potential roles in myoblasts proliferation. The genes in cluster K4 were significantly enriched embryonic skeletal muscle morphogenesis, consistent with the process of myotubes development.^55^, ^56^ Notably, skeletal myogenesis showed high dynamic in cluster K2 and cluster K3, reflecting the peak of myogenesis progress. Taken together clustering of gene expression profiles demonstrated that each cluster comprised unique gene expression pattern may reveal time‐dependent modules of transcriptional regulation during skeletal muscle development.

### 3.3 Dynamics of the chromatin accessibility and TF regulatory network for bovine skeletal muscle development

**FIGURE 3:**
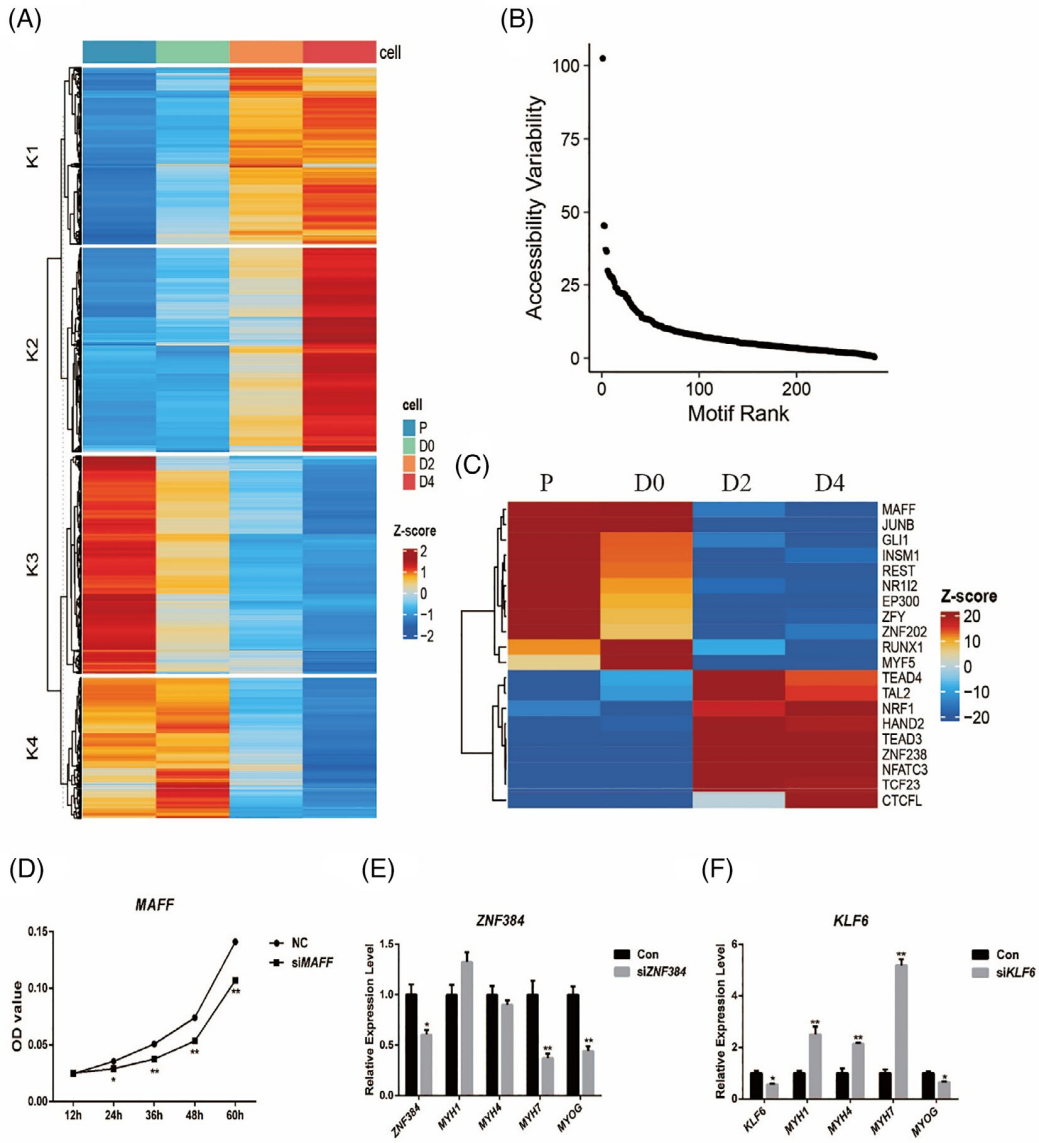
Dynamic of chromatin landscape and TF regulatory during myogenesis. (A) Heatmap of differentially accessible chromatin regions, yielding four clusters of co‐regulated peak sets. (B) Rank order plot of the transcription factor variability in the OCRs during myogenesis. (C) Heatmap displaying the top 20 most variably motifs. (D) Cell proliferation was measured by CCK8 assay of expressing si*MAFF*. (E, F) Relative gene expression of muscle‐specific genes (*MYH1*, *MYH4*, *MYH7* and *MYOG*) following siRNA knockdown of *ZNF384* and *KLF6*. *p < 0.05, **p < 0.01

We next sought to investigate dynamic changes in chromatin accessibility during bovine myoblasts proliferation and myogenic differentiation. We detected a total of 42,211 differentially accessible chromatin regions (DARs) among each pairwise comparison, which clustered into four groups via k‐means clustering method. Dramatic and widespread changes in chromatin accessibility were evident at four differentiated time points, which exhibited a similar profile with gene expression profile (Figure 3A). Remarkably, we found that massive differential accessibility regions occurred at P and D4 time points, and less appeared at D0 and D2 time points, supporting changing and stable chromatin accessibility states during bovine skeletal muscle differentiation. Cluster K3 was comprised of 12,421 open chromatin regions (OCRs) that activated at P time point, and became less accessible at the later stages. In contrast, cluster K2 was composed of 11,652 OCRs that displayed relatively closed chromatin accessible at the early stages, and activated at D4 time point. Cluster K4 consisted of 8033 OCRs gained accessible during the whole period of myoblasts proliferation, while cluster K1 contained 10,105 OCRs remaining accessible during the whole period of myoblast proliferation.

Transcription factors and their regulatory programs mediate the key developmental events in myoblasts proliferation and differentiation. Here, we applied chromVAR to dissect transcription factor variability in the OCRs at four time points (Figures 3B,C and Data S1). Among the most variably accessible sequence motifs determined, we identified important TF motifs such as *MAFF*, *JUNB*, *Runx1*, *MYF5*, *TEAD3* and *TEAD4*. Many TFs from the group were directly related to skeletal muscle cell proliferation and differentiation, and their transcription regulation activity was consistent with their relevant regulatory roles.^57^, ^58^, ^59^, ^60^ Combined with differential chromatin accessibility, we observed profound loss of chromatin accessibility at promotors region of *NR1I2* (Figure S3A). Although we found *MAFF*, *ZNF384* and *KLF6* exhibited dynamic activity in four time points (Figure S3B), their roles in bovine myoblasts proliferation or differentiation have not been reported. We investigated their effects on myogenesis in cell models using RNA interference. Myoblasts proliferation was inhibited after *MAFF* silencing (Figure 3D). Silencing *ZNF384* impaired *MYOG* and *MYH7* expression, while silencing *KLF6* improved the *MYH1*, *MYH4* and *MYH7* expression but inhibited *MYOG* expression (Figure 3E,F). These findings suggest an integrative role for these factors in the regulatory mechanisms of bovine myoblasts proliferation and myogenic differentiation.

### 3.4 Stage‐specific gene expression during bovine myoblasts proliferation and myogenic differentiation

**FIGURE 4:**
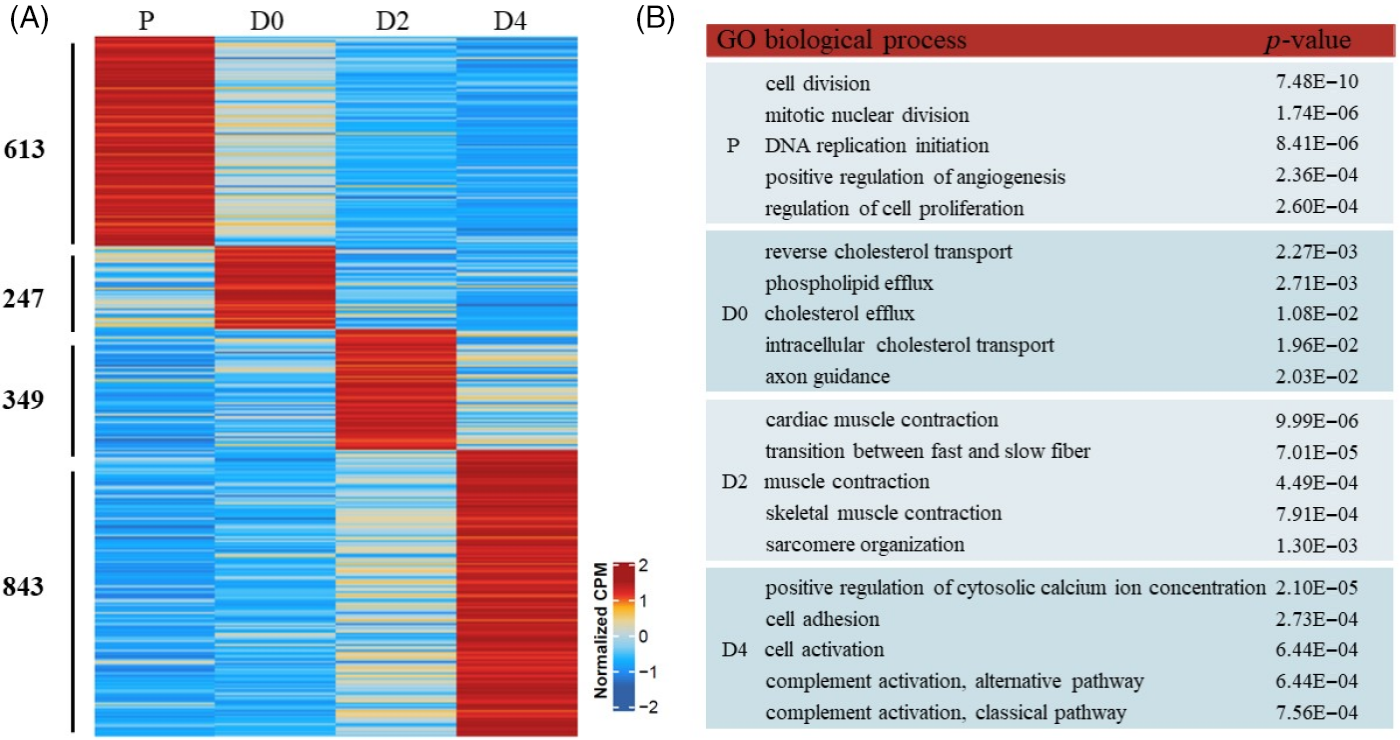
Characterization of stage‐specific genes. (A) Heatmap of stage‐specific genes identified at each time point. (B) Table showing the top five GO terms enriched at P, DO, D2 and D4, respectively. Only GO terms for GOTERM_BP_DIRECT and *p*‐value < 0.05 were considered

To characterize the persistent and stage‐specific genetic mechanisms underlying skeletal muscle myogenesis, we next identified gene sets that temporally regulated at different time points. Using Shannon‐entropy‐based method, we identified 613, 247, 349 and 843 stage‐specific genes at P, D0, D2 and D4 time point, respectively (Figure 4A). To further investigate whether specific genes at each time point have a specific gene regulatory pattern, we applied GO enrichment analysis (Figure 4B). We observed that the GO terms of specific genes at each time point were different from those of differentially expressed genes to some extent. Stage‐specific genes at P and D2 time points were enriched for GO terms related to cell division and skeletal muscles development, respectively. Interestingly, we found distinct GO terms enriched in D0 and D4 time points. Reverse cholesterol transport (*p* <2.27 × 10^−3^), cholesterol efflux (*p* <1.08 × 10^−2^) and intracellular cholesterol transport (*p* <1.96 × 10^−2^) were significantly enriched in stage‐specific genes at D0 time point, while positive regulation of cytosolic calcium ion concentration (*p* <2.10 × 10^−5^), cell adhesion (*p* <2.73 × 10^−4^) and cell activation (*p* <6.44 × 10^−4^) were significantly enriched in stage‐specific genes at D4 time point. Altogether, stage‐specific genes expression at each time point appears to establish a key restriction point for bovine myoblasts proliferation and myogenic differentiation.

### 3.5 Stage‐specific open chromatin reveals distinct TF binding events during bovine myoblasts proliferation and myogenic differentiation

**FIGURE 5:**
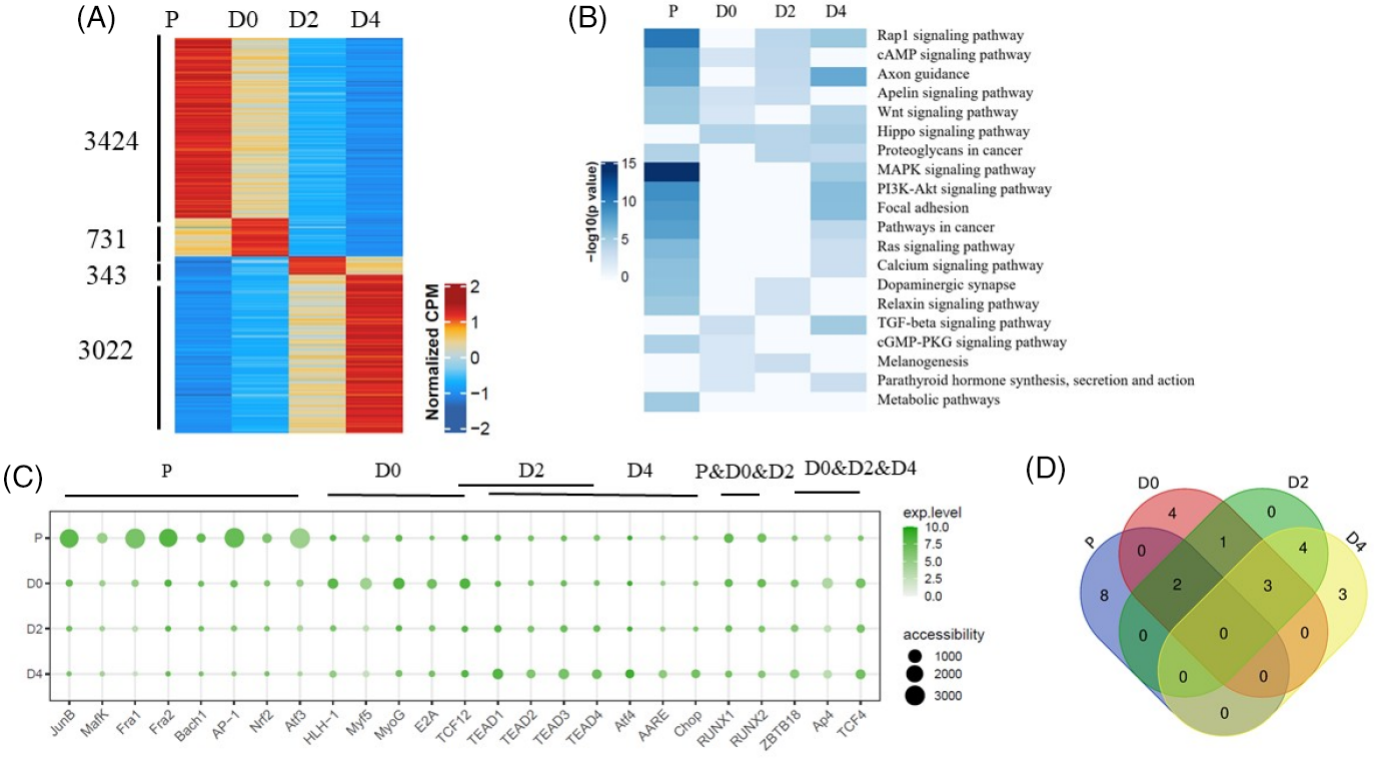
Characterization of stage‐specific peaks. (A) Heatmap of stage‐specific peaks identified at each time point. (B) Heatmap displaying the top 20 significantly enriched KEGG pathways across four time points. *p*‐value < 0.05 is statistically significant. (C) The top 10 known TF binding motifs enrichment within stage‐specific accessible regions. The dot colour represents gene expression level of TFs and the dot size represents the motif‐enrichment value identified by HOMER (−log *p* value). (D) Veen diagram showing the overlap of top 10 known TF binding motifs enrichment at each time point

As lineage commitment and myogenic differentiation relies on the activity of specific transcriptional programs, we asked whether the stage‐specific ATAC‐seq peaks harbour specific TFs regulating skeletal muscle development. A total of 3424, 731, 343 and 3022 stage‐specific peaks were identified at P, D0, D2 and D4 time points, respectively (Figure 5A). Interestingly, we observed a progressive increase in the number of stage‐specific peaks at P and D4 time points and the majority of stage‐specific peaks fell into enhancer regions (distal intergenic and intronic regions; Figure S4). The enriched GO terms were involved in a variety of biological functions, while only a small part of GO terms was directly related with skeletal muscle growth and development (Figure S5). The enriched pathways were Rap1, cAMP, Wnt, Hippo and MAPK signalling pathways, which were associated with muscle growth and development (Figure 5B). Next, we investigated the characters of TF binding in stage‐specific peaks. Notably, we revealed a series of TFs that showed specific enrichment at different time points (Figure 5C). We observed specific enrichment motif of the *ATF3*, *AP‐1* and its family members (*FRA‐1*, *FRA‐2*, *JUNB*) at P time point, which is in accordance with the observation that *AP‐1* complex is correlated with cell proliferation and transformation.^61^, ^62^ We also found that high motif enrichment of *MyoG*, *Myf5*, *HLH‐1* appeared at D0 time and these TFs have previously been shown to be markers of muscle lineage specification,^63^, ^64^, ^65^ suggesting that the differentiation program may be initiated at this stage. It is important to note that the TFs, *ZBTB18*, *TCF4*, *TEAD1*, *TEAD2*, *TEAD3* and *TEAD4*, enriched between D2 and D4 time point were largely consistent (Figure 5D), suggesting a continuous regulatory mechanism that modulates myogenic differentiation.

### 3.6 Integrative analysis of chromatin accessibility and gene expression

**FIGURE 6:**
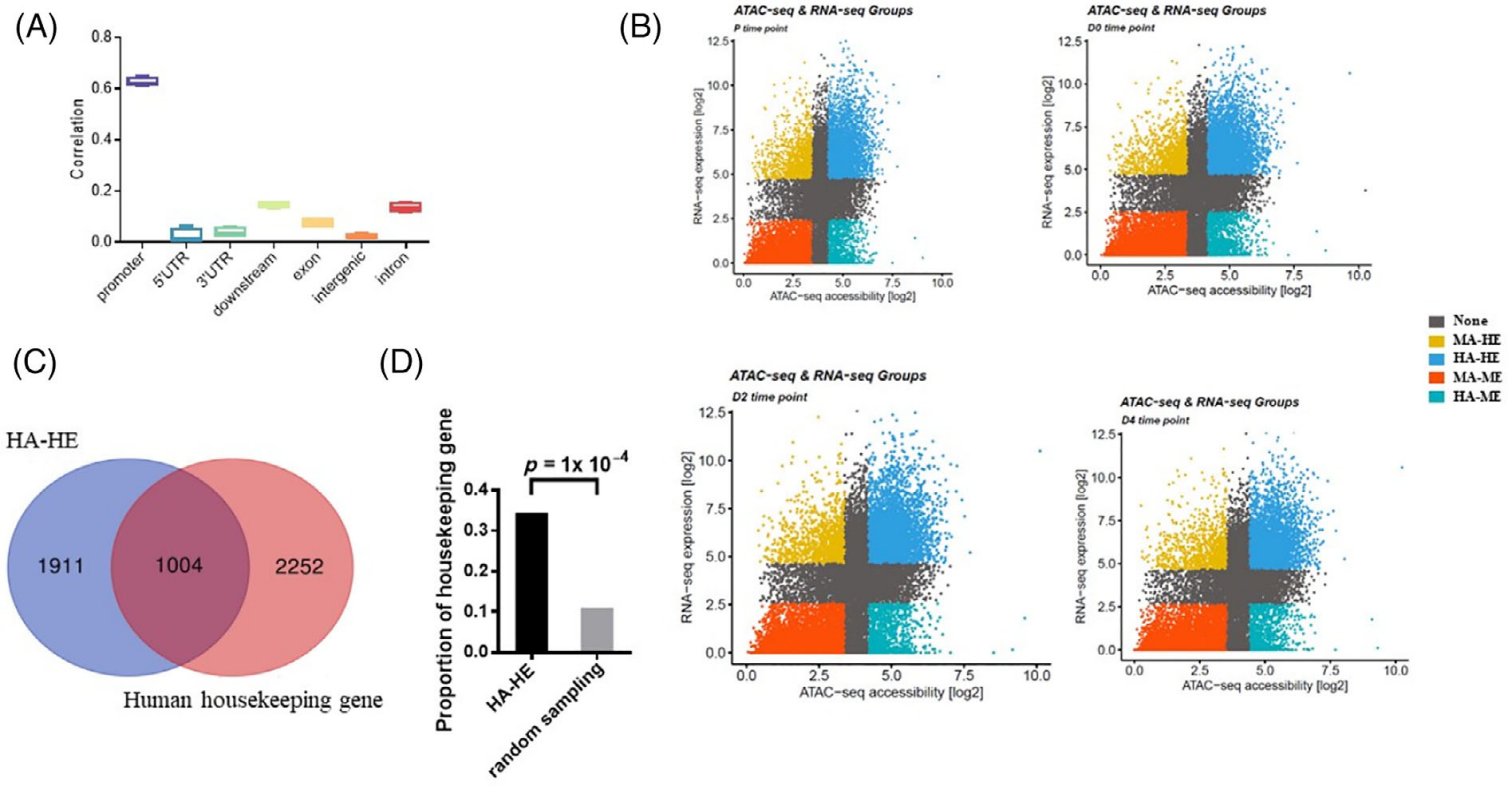
Chromatin accessibility at promoter positively correlates with gene expression. (A) Boxplot of the Spearman correlation between normalized counts of RNA‐seq (log2CPM) and normalized counts of ATAC‐seq (log2CPM) at promoter (TSS ± 2.5 kb), 5′UTR, 3′UTR, downstream, exon, intergenic and intron. (B) Scatter plot showing positive relationships between gene expression level and chromatin accessibility level at promoter for P, D0, D2 and D4 time points. Based on the level of gene expression and chromatin accessibility, genes are divided into four groups: HA–HE: high accessibility/high expression; MA–ME: medium‐low accessibility/medium‐low expression; HA–ME: high accessibility/medium‐low expression; MA–HE: medium‐low accessibility/high expression. (C) Veen diagram showing the overlap between HA–HE genes and bovine orthologous housekeeping genes. (D) Enrichment analysis of HA–HE genes in bovine orthologous housekeeping genes compared to random genes. Random selections of 2915 genes from all protein‐coding genes

Enhancer and promoter regions are key players in gene expression regulation through transcription initiation (promoters) and by amplifying such transcription initiation (enhancers).^66^, ^67^, ^68^, ^69^ Overall, we observed only chromatin accessibility at promoter was positively correlated with its gene expression [Spearman's *R* (promoter) = 0.62], whereas the correlation at other genomic regions was relatively weak (Spearman's *R* <0.14; Figure 6A). To enhance our understanding of the positive relationship, we further divided genes into groups according to their promoter accessibility and expression levels (Figure 6B). As in previous reports, genes with both high expression level and accessibility at promoter region were enriched for housekeeping‐like biological functions.^43^, ^70^ Therefore, we verified whether this regulatory pattern was also present during bovine skeletal muscle development. In general, we found a significantly higher proportion (proportion = 34.42%, *p*‐value <10^−4^) of HA‐HE genes were bovine orthologous housekeeping genes (Figure 6C,D). In summary, our results indicated a potential relationship between chromatin accessibility and gene expression, which is paramount to understanding the complexities of gene regulatory during bovine skeletal muscle development.

### 3.7 Cattle growth‐related genetic variants in the context of chromatin dynamics

**FIGURE 7:**
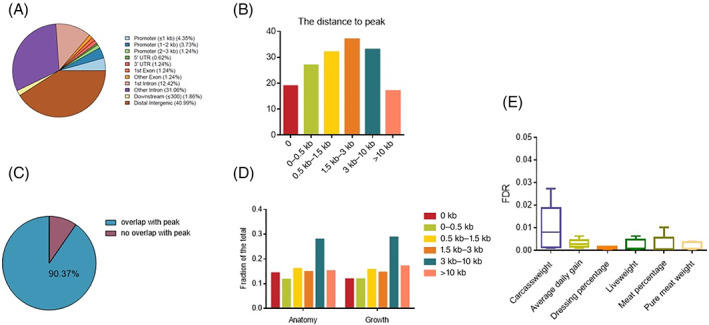
Cattle growth‐related genetic variants associated with chromatin accessibility regions. (A) Genomic distribution of 163 stature‐associated lead SNPs identified by Aniek C. Bouwman et al. (B) The distribution of the distance between 163 stature‐associated lead SNPs and their nearest OCRs. Peak distance is grouped into 6 categories: 0 kb (located within the peak), 0–0.5, 0.5–1.5, 1.5–3, 3–10 and >10 kb. (C) The overlap between 163 stature‐associated QTL confidence regions and OCRs. (D) Distribution of the distance between known QTL regions and their nearest OCRs. The known QTL regions and associated SNP for anatomy and growth traits are obtained from cattle QTLdb. (E) Boxplot showing the enrichment of GWAS signals for chromatin accessibility obtained from myoblasts proliferation peaks (cluster K3 and K4), myogenic differentiation peaks (cluster K1 and K2), stage‐specific peaks, DARs peaks and OCRs peaks. The x‐axis represents growth traits, including carcass weight, average daily gain, dressing percentage, liveweight, meat percentage and pure meat weight. The *y*‐axis represents FDR (adjusted *p* values) obtained from enrichment analysis

To dissect genetic aspects of skeletal muscle development, we further investigated whether the open chromatin regions dynamics were associated with muscle‐related traits. In this study, we first investigated the association between our data and 163 stature‐associated lead SNPs identified by Bouwman et al.^71^ (Figure 7A). Although very few of them were located in accessible chromatin peaks, the majority of these lead variants were located within 3 kb of OCRs (Figure 7B). There were 11.5% of lead variants located in OCRs, while 70.55% of lead variants were located within 3 kb. Since there is a reasonable chance that the lead SNP is not the causal variant, we also examined the fractions of OCRs that overlapped with QTL confidence regions and found that a great number (90.37%) of QTL confidence regions were overlapped with at least one OCRs (Figure 7C). A similar pattern was observed in context from cattle QTLdb. About 56.92% and 54.09% of known QTL regions and associated SNP located within 3 kb of OCRs for anatomy and growth traits, respectively (Figure 7D, Table S5). Thus, we expect that the joint effect of genetic variations in OCRs might impact cattle growth‐related traits. We categorized five groups: myoblasts proliferation peaks (cluster K3 and K4), myogenic differentiation peaks (cluster K1 and K2), stage‐specific peaks, DARs peaks, OCRs peaks. Significant enrichments (FDR <0.05) were observed for carcass weight, average daily gain, dressing percentage, liveweight, meat percentage and pure meat weight in all five chromatin peak groups. (Figure 7E, Table S6). Notably, we found the joint SNP effects in OCRs were lower than those in other groups (Table S6). Given that the majority of genetic variants fell into non‐coding regions, our data therefore may be suitable for the functional interpretation of GWAS results.

## 4 DISCUSSION

Previous studies have provided powerful evidence for the crucial roles of cis‐regulatory elements (CREs) in identifying causative variants of complex traits.^16^, ^72^, ^73^ With driven efforts by FAANG, a growing body of studies engaged in the functional annotation of farm animal genome have emerged, further leading to characterize the genetic architecture of important economic traits and enhance the animal performance.^20^, ^22^, ^74^, ^75^ Although major advances have been made in bovine epigenome study, the epigenetic basis for foetal skeletal muscle development remains poorly understood. An important limitation for this study is the lack of ideal samples across multiple developmental time points of bovine skeletal muscle development. In our study, we recapitulated the time course of foetal bovine myoblasts proliferation and myogenic differentiation, which is an ideal model to study bovine early skeletal muscle development *in vitro*. Furthermore, we inferred the gene expression and transcription regulator dynamic change, investigated the major genes that exhibit maximum activity, and identified potential TFs motif that acts actively in accessibility peaks. As a result, the chromatin accessibility and gene expression profiles between time points exhibited widespread changes and period specificity. We comprehensively characterized co‐expressed genes clusters and highly variable TFs during bovine myoblasts proliferation and myogenic differentiation, providing a novel insight to capture key transitions and turning points during skeletal muscle development. CREs play essential roles in development, whereas promoter and enhancer serve as the best understood types of CREs.^76^, ^77^, ^78^ Notably, chromatin accessibility across the genome reflects specific binding events at CREs. Despite chromatin accessibility is a powerful marker of active regulatory elements, alone it provides only a partial view of the regulatory mechanisms underlying skeletal muscle development. Further investigations with additional epigenetic data, such as histone modification, are still necessary for accurate identification of CREs in the future.

In order to characterize the sequence of developmental steps leading to bovine myoblasts proliferation and myogenic differentiation, we applied the Shannon‐entropy‐based method to capture specification gene expression pattern and TF binding events. In terms of specific gene pattern, enrichment for GO terms at the early stages of myoblasts proliferation and myogenic differentiation was expected, while GO terms enriched at the later stages exhibited distinct biological activities, denoting their specific and temporal roles. The later stages of myoblasts proliferation enriched cholesterol‐related GO terms such as reverse cholesterol transport, cholesterol efflux and intracellular cholesterol transport, and the later stages of myogenic differentiation enriched GO terms associated with positive regulation of cytosolic calcium ion concentration, cell adhesion and cell activation. Notably, these biological processes were not observed in differentially expressed genes from each pairwise comparison, elucidating that the stage‐specific expression may be responsible for the specific molecular mechanisms. During skeletal muscle development, myoblasts undergo a number of biochemical and morphological changes, including membrane cholesterol decreased during the early stages of myoblasts fusion.^79^, ^80^, ^81^, ^82^, ^83^ For instance, previous studies had demonstrated that *ABCG1*‐ and *ABCA1*‐mediated cholesterol efflux played an important role in reducing cell plasma membrane cholesterol content and apoA‐IV encoded by *APOA4* gene is known to participate in promoting cholesterol efflux.^84^, ^85^, ^86^, ^87^, ^88^ In addition, elevation in cytosolic calcium, membrane remodelling and adhesive interactions are considered to participate in the fusion of myoblasts into multinucleated myotubes.^89^, ^90^, ^91^, ^92^, ^93^, ^94^ Alternatively, these stage‐specific genes may contribute to bovine myoblasts proliferation and myogenic differentiation by mediating these biological processes. Overall, these results further supported our prediction on the functions of stage‐specific genes, suggesting the reliability of our data analysis. As for nearby genes of stage‐specific peaks, we found significant decreases in the number of GO terms related to myogenesis, although muscle growth and development such as Rap1, cAMP, Wnt, Hippo and MAPK signalling pathways were identified. Indeed, the investigation on gene regulatory mechanisms regulated by stage‐specific peaks in our study may be limited on the assumption that genes are regulated by proximal regulatory elements. It must be noted that this assumption is not always correct. Additionally, a series of TF binding events occurred as expected during bovine myoblasts proliferation and myogenic differentiation, including *ATF3*, *AP‐1* family members, *MyoG*, *Myf5*, *HLH‐1*, *ZBTB18*, *TCF4* and *TEAD* family. We propose that these TFs may facilitate our understanding of the sequential order of gene regulatory during skeletal muscle growth and development.

Previous studies have pointed to a strong relationship between gene expression and chromatin accessibility at promoter region.^43^, ^95^ However, given the genetic basis of complex cis‐regulatory mechanisms, the relationship between chromatin accessibility and gene expression remains functionally uncharacterized at present. In this work, chromatin landscape was classified into six groups based on their genomic regions, and relationships were calculated between chromatin accessibility and gene expression in each group. We only observed a strong positive relationship between chromatin accessibility and their nearby gene expression in promoter group, which were consistent with the previous study.^43^Transcriptional regulation is governed not only by proximal cis‐regulatory elements (promoter), but also by distal cis‐regulatory elements (enhancer) that often located far away from their target gene.^96^ While such a strong positive relationship was absent in intergenic group, this is partly due to a remarkably large amount of intergenic regions widely distributed across the genome and the inaccurate method of nearby genes annotation. Furthermore, we provided evidence showing that at least one‐third of the genes (high expression and high accessibility level at promoter region) were correlated with housekeeping genes. Considering the potential significance of the residual HA/HE genes, we suppose that this regulatory model is an essential step towards completing our understanding of the regulatory mechanism of skeletal muscle development.

Genome‐wide association studies (GWAS) have proved to be an effective and promising approach for the identification of genetic variants associated with complex traits.^97^ However, most of these genetic variants are located in non‐coding regions and a push for annotation of cis‐regulatory elements by epigenomic information have contributed to elucidate the functional relevance of non‐coding genetic variants to some extent.^98^ In this study, integration of chromatin landscape profiles with GWAS signals demonstrated that the GWAS signals of bovine growth traits were significantly enriched in chromatin accessibility of skeletal muscle development, which elucidated the activity of genetic variants in skeletal muscle development‐related cis‐regulatory elements. Further studies are warranted to prioritize variant SNPs and explain how these variant SNPs change TFs regulatory and genes expression.

## 5 CONCLUSION

Overall, our study describes the dynamic changes of chromatin accessibility and gene expression during bovine myoblasts proliferation and differentiation in vitro, which is a good model to reconstruct the process of skeletal muscle development. In addition, we identified a series of stage‐specific genes and TFs to enhance our understanding of the sequential regulation of skeletal muscle development. Integrations of chromatin accessibility with transcriptional expression profiles and GWAS signals provide an opportunity to explore the regulatory role of these cis‐regulatory elements defined by chromatin accessibility. Taken together, our study demonstrates a step‐wise dissection of the transcriptional regulation network for skeletal muscle development and provide a systematic understanding of the molecular circuits governing skeletal muscle development.

## CONFLICT OF INTEREST

The authors declare that the research was conducted in the absence of any commercial or financial relationships that could be construed as a potential conflict of interest.

## AUTHOR CONTRIBUTIONS

Qian Li and Yahui Wang performed the experiments, analysed the data and drafted the manuscript. Junya Li and Lupei Zhang conceived and supervised the experiments. Xin Hu, Yapeng Zhang, Hongwei Li, and Qi Zhang participated in data collection and analysis. Wentao Cai, Zezhao Wang, Bo Zhu, Lingyang Xu, Xue Gao, Yan Chen, and Huijiang Gao contributed to data analysis and manuscript preparation.

## ETHICS STATEMENT

All experiments were conducted in concordance with the guidelines established by the Regulations for the Administration of Affairs Concerning Experimental Animals (Ministry of Science and Technology, China, 2004). All animal experimental protocols were approved by the Animal Ethics Committee of the Institute of Animal Sciences, Chinese Academy of Agricultural Sciences (No. IAS2021‐52). Gravid cows were raised by Jingxinxufa Agriculture Co., Ltd. (Weichang, China), and all efforts were made to minimize suffering.

## Supporting information


**Data S1** The ranked list displaying TF motifs activity during bovine myoblasts proliferation and myogenic differentiation.

**Data S2** Stage‐specific peaks identified during bovine myoblasts proliferation and myogenic differentiation.

**Table S1** Primers sequence for real‐time quantitative PCR.
**Table S2:** Target sequences of siRNA.
**Table S3:** Summary of the ATAC‐seq data for each replicate.
**Table S4:** Summary of the RNA‐seq data for each replicate.
**Table S5:** Summary of cattle anatomy and growth‐related traits obtained from Cattle QTLdb.
**Table S6:** GWAS enrichment results for cattle carcass weight, average daily gain, dressing percentage, meat percentage and pure meat weight traits.
**Figure S1**: Library evaluation of ATAC‐seq and RNA‐seq profiles. (A) ATAC‐seq fragment size distribution with a distinct nucleosomal pattern (mono‐, di‐, and tri‐nucleosomes) for each replicate. The x‐axis represents the fragment size distribution and the *y*‐axis represents the abundance. (B) the expression levels of major myogenic regulatory factors (*MyoD*, *Myf5*, *MyoG* and *Myf6*) at P, D0, D2 and D4 time point.
**Figure S2**: Quality assessment of ATAC‐seq and RNA‐seq profiles. (A, B) Heatmap displaying the Spearman correlation coefficient of normalized RNA‐seq reads (all genes) and normalized ATAC‐seq reads (top 50,000 open chromatin peaks) between each pairwise comparison. (C) Normalized coverage (CPM) of chromatin accessibility and mRNA levels around *MyoG*, *Myf5* and *Myf6* genes for each time points.
**Figure S3**: TF regulatory during bovine myoblasts proliferation and myogenic differentiation. (A) Normalized coverage (CPM) of chromatin accessibility around *NR1I2* loci for each time point. (B) Heatmap displaying the top 40 most variably motifs.
**Figure S4:** Genomic distribution of stage‐specific peaks at P, D0, D2 and D4 time point.
**Figure S5**: GO term biological function governed by stage‐specific peaks. Top GO terms (GOTERM_BP_DIRECT, GOTERM_CC_DIRECT and GOTERM_MF_DIRECT) for stage‐specific peaks annotated to nearby genes at P, D0, D2 and D4 time point, respectively. *p*‐value < 0.05 is statistically significant.

## Data Availability

All relevant datasets generated in this study have been deposited in a NCBI SRA database with accession PRJNA790762. Processed data files are available in Supplementary Data.
