# Supplementary material for: Transcriptional states and chromatin accessibility during bovine myoblasts proliferation and myogenic differentiation
Source: Cell Prolif. 2022 Apr 1;55(5):e13219. doi: 10.1111/cpr.13219 (PMC9136495; doi:10.1111/cpr.13219)
Supplement: Supplementary file 3 — Table S1 Primers sequence for real‐time quantitative PCR. Table S2: Target sequences of siRNA. Table S3: Summary of the ATAC‐seq data for each replicate. Table S4: Summary of the RNA‐seq data for each replicate. Table S5: Summary of cattle anatomy and growth‐related traits obtained from Cattle QTLdb. Table S6: GWAS enrichment results for cattle carcass weight, average daily gain, dressing percentage, meat percentage and pure meat weight traits. Figure S1: Library evaluation of ATAC‐seq and RNA‐seq profiles. (A) ATAC‐seq fragment size distribution with a distinct nucleosomal pattern (mono‐, di‐, and tri‐nucleosomes) for each replicate. The x‐axis represents the fragment size distribution and the y‐axis represents the abundance. (B) the expression levels of major myogenic regulatory factors (MyoD, Myf5, MyoG and Myf6) at P, D0, D2 and D4 time point. Figure S2: Quality assessment of ATAC‐seq and RNA‐seq profiles. (A, B) Heatmap displaying the Spearman correlation coefficient of normalized RNA‐seq reads (all genes) and normalized ATAC‐seq reads (top 50,000 open chromatin peaks) between each pairwise comparison. (C) Normalized coverage (CPM) of chromatin accessibility and mRNA levels around MyoG, Myf5 and Myf6 genes for each time points. Figure S3: TF regulatory during bovine myoblasts proliferation and myogenic differentiation. (A) Normalized coverage (CPM) of chromatin accessibility around NR1I2 loci for each time point. (B) Heatmap displaying the top 40 most variably motifs. Figure S4: Genomic distribution of stage‐specific peaks at P, D0, D2 and D4 time point. Figure S5: GO term biological function governed by stage‐specific peaks. Top GO terms (GOTERM_BP_DIRECT, GOTERM_CC_DIRECT and GOTERM_MF_DIRECT) for stage‐specific peaks annotated to nearby genes at P, D0, D2 and D4 time point, respectively. p‐value < 0.05 is statistically significant. [file CPR-55-e13219-s002.docx]

Supplementary Material

**Supplementary Tables**

**Table S1: Primers sequence for real-time quantitative PCR.**

| Gene | Forward Primer Sequence（5'→3'） | Reverse Primer Sequence（5'→3'） |
| --- | --- | --- |
| *18S* | GTAACCCGTTGAACCCCATT | CCATCCAATCGGTAGTAGCG |
| *MAFF* | GTGTGCCAGAAGGAGGAGC | CCGACTTGACGATAGTGATGAC |
| *ZNF384* | ACTACCCCACCTTGCTGACA | TTGACTCCCTTCTCTTCTCCA |
| *KLF6* | CCCTGGAGGAATACTGGCA | CGAGTTCAGGCTGTTGGTCT |
| *MYH1* | GGGAAACTGGCTTCTGCTGAT | TGGGTTGGTGGTGATTAGGAG |
| *MYH4* | CTCCTAATCACCACCAACCCATA | TGTCAGCAACTTCAGTGCCATC |
| *MYH7* | AAGACAGTGACCGTGAAGGAGG | GGTTGATGGTGACGCAGAAGA |
| *MYOG* | CCGTGGGCGTGTAAGGTGTG | CCTCTGGTTGGGGTTGAGCAG |

**Table S2: Target sequences of siRNA.**

| Sequence name | Target sequence（5'→3'） |
| --- | --- |
| si-*MFFF*_001 | GCAAAGCGCTGAAGATCAA |
| si-*MAFF*_002 | ATCACTATCGTCAAGTCGG |
| si-*MAFF*_003 | TGCAGAAGCAGAAGTCGGA |
| si-*ZNF384*_001 | CGGAAACGTACCTGATGAA |
| si-*ZNF384*_002 | GAGCATCACAAGGACATCT |
| si-*ZNF384*_003 | GGTTTGCTATGACCTCATA |
| si-*KLF6*_001 | CAGGAAGATCTGTGGACCAAA |
| si-*KLF6*_002 | CAGGAAAGTTTACACCAAA |
| si-*KLF6*_003 | TACAACCTGGAGACCAACA |

**Table S3: Summary of the ATAC-seq data for each replicate**. Total raw reads, the number and ratio of mapped reads, the number and ratio of filter reads (high mapping quality, non-duplicate, nonmitochondrial uniquely mapping), the number of peaks called by MACS2.

| **Sample** | **Total Reads** | **Aligned Reads**  **(%Total)** | **Filtered reads,**  **(%Total)** | **Peak number** |
| --- | --- | --- | --- | --- |
| P-Rep1 | 101499018 | 97254286  (95.82%) | 61293932  (60.39%) | 257095 |
| P-Rep2 | 100018586 | 96506852  (96.49%) | 62512760  (62.50%) | 261858 |
| D0-Rep1 | 107173412 | 102793118  (95.91%) | 65031214  (60.68%) | 275311 |
| D0-Rep2 | 116707806 | 112956740  (96.79%) | 75034522  (64.29%) | 309729 |
| D2-Rep1 | 100820920 | 97423612  (96.63%) | 63758370  (63.24%) | 225975 |
| D2-Rep2 | 94676646 | 88880002  (93.88%) | 50524228  (53.37%) | 192928 |
| D4-Rep1 | 91685362 | 87367338  (95.29%) | 57087112  (62.26%) | 247724 |
| D4_Rep2 | 94200728 | 89570304  (95.08%) | 54905486  (58.29%) | 241907 |

**Table S4: Summary of the RNA-seq data for each replicate.** Total raw reads, the number and ratio of mapped reads with high mapping quality, the number of reads assigned to gene.

| **Sample** | **Total Reads** | **Aligned Reads**  **(%Total)** | **Reads Assigned to Genes**  **(%Total)** |
| --- | --- | --- | --- |
| P-Rep1 | 20361793 | 19728360  （96.89%） | 16662529  （81.83%） |
| P-Rep2 | 23009732 | 22281905  （96.84%） | 19348935  （84.09%） |
| D0-Rep1 | 21253728 | 20599384  （96.92%） | 17355305  （81.66%） |
| D0-Rep2 | 22878864 | 22195588  （97.01%） | 18781818  （82.09%） |
| D2-Rep1 | 25041900 | 24293403  （97.01%） | 20727090  （82.77%） |
| D2-Rep2 | 21215765 | 20625216  （97.22%） | 17281684  （81.46%） |
| D4-Rep1 | 22987375 | 22264931  （96.86%） | 18896009  （82.20%） |
| D4_Rep2 | 21025258 | 20374404  （96.90%） | 17064612  （81.16%） |

**Table S5: Summary of cattle anatomy and growth-related traits obtained from Cattle QTLdb.**

| **Anatomy traits** | **Growth traits** |
| --- | --- |
| Carcass length  Carcass weight  Longissimus muscle area  Longissimus muscle area growth  Longissimus muscle area/muscle fiber area  Longissimus muscle lean area  Longissimus muscle length  Longissimus muscle weight  Longissimus muscle width  Muscle fiber area  Muscle fiber diameter  Semispinalis lean area  Semitendinosus muscle weight  Sirloin weight | Average daily gain  Body capacity  Body depth  Body length  Body length (birth)  Body size  Body weight  Body weight (6, 18, 24 months)  Body weight (600 days)  Body weight (birth, initial, weaning, yearling, at castration, mature, mean, slaughter, test end)  Body weight gain  Growth index  Height (200, 400 days)  Height (6 months, yearling, mature, 24 months)  Longissimus muscle area growth  Metabolic body weight  Percentage decrease in body weight up to day 150 after challenge  Withers height |

**Table S6: GWAS enrichment results for cattle carcass weight, average daily gain, dressing percentage, meat percentage and pure meat weight traits.** CW, carcass weight; DG, average daily gain; DP, dressing percentage; LW, liveweight; MP, meat percentage; PM, pure meat weight.

| Traits | myoblasts  proliferation | | | myogenic  differentiation | | | stage-specific | | | DARs | | | OCRs | | | |
| --- | --- | --- | --- | --- | --- | --- | --- | --- | --- | --- | --- | --- | --- | --- | --- | --- |
|  | p-value | FDR | Fold | p-value | FDR | Fold | p-value | FDR | Fold | p-value | FDR | Fold | *p*-value | FDR | Fold |  |
| CW | 0.0010 | 0.0017 | 2.03 | 0.0272 | 0.0272 | 1.96 | 0.0072 | 0.0080 | 1.98 | 0.0101 | 0.0104 | 1.98 | 0.0005 | 0.0011 | 1.74 |  |
| DG | 0.0018 | 0.0027 | 2.04 | 0.0019 | 0.0027 | 2.08 | 0.0055 | 0.0063 | 2.02 | 0.0005 | 0.0011 | 2.05 | 0.0014 | 0.0022 | 1.78 |  |
| DP | 0.0009 | 0.0016 | 2.03 | 0.0008 | 0.0015 | 2.07 | 0.0008 | 0.0015 | 2.08 | 0.0004 | 0.0011 | 2.03 | 0.0001 | 0.0005 | 1.77 |  |
| LW | 0.0002 | 0.0007 | 2.06 | 0.0055 | 0.0063 | 2.02 | 0.0025 | 0.0033 | 2.06 | 0.0003 | 0.0009 | 2.04 | 0.0001 | 0.0005 | 1.79 |  |
| MP | 0.0001 | 0.0005 | 2.09 | 0.0094 | 0.0101 | 2.00 | 0.0005 | 0.0011 | 2.05 | 0.0001 | 0.0005 | 2.02 | 0.0001 | 0.0005 | 1.78 |  |
| PM | 0.0031 | 0.0039 | 1.98 | 0.0002 | 0.0007 | 2.07 | 0.0024 | 0.0033 | 2.03 | 0.0003 | 0.0009 | 2.02 | 0.0001 | 0.0005 | 1.77 |  |

**Supplementary Figures**

**
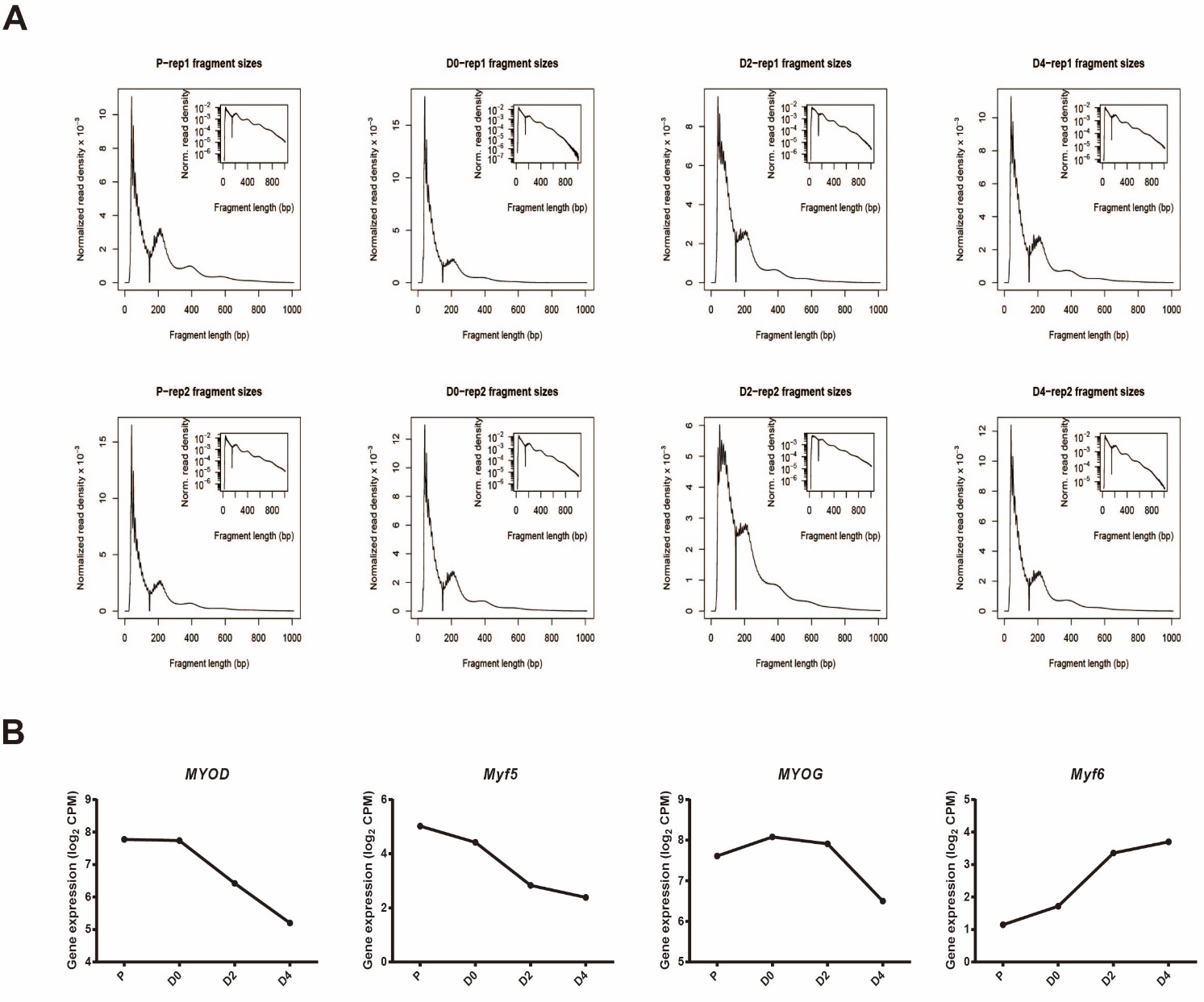
Supplementary Figure 1: Library evaluation of ATAC-seq and RNA-seq profiles.** (A) ATAC-seq fragment size distribution with a distinct nucleosomal pattern (mono-, di-, and tri-nucleosomes) for each replicate. The x-axis represents the fragment size distribution and the y-axis represents the abundance. (B) the expression levels of major myogenic regulatory factors (*MyoD*, *Myf5*, *MyoG* and *Myf6*) at P, D0, D2 and D4 time point.


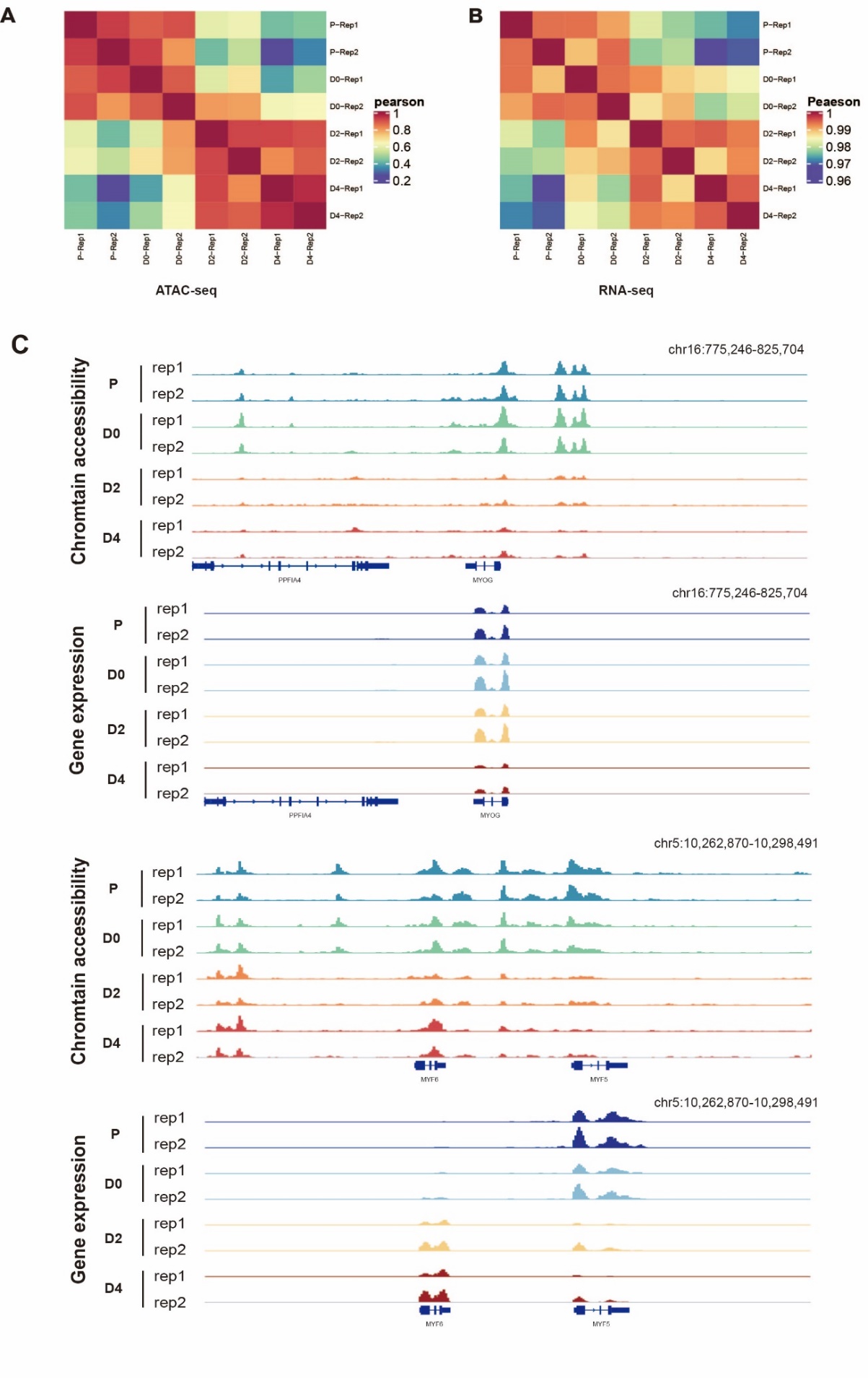


**Supplementary Figure 2: Quality assessment of ATAC-seq and RNA-seq profiles.** (A, B) Heatmap displaying the Spearman correlation coefficient of normalized RNA-seq reads (all genes) and normalized ATAC-seq reads (top 50,000 open chromatin peaks) between each pairwise comparison. (C) Normalized coverage (CPM) of chromatin accessibility and mRNA levels around *MyoG*, *Myf5* and *Myf6* genes for each time points.


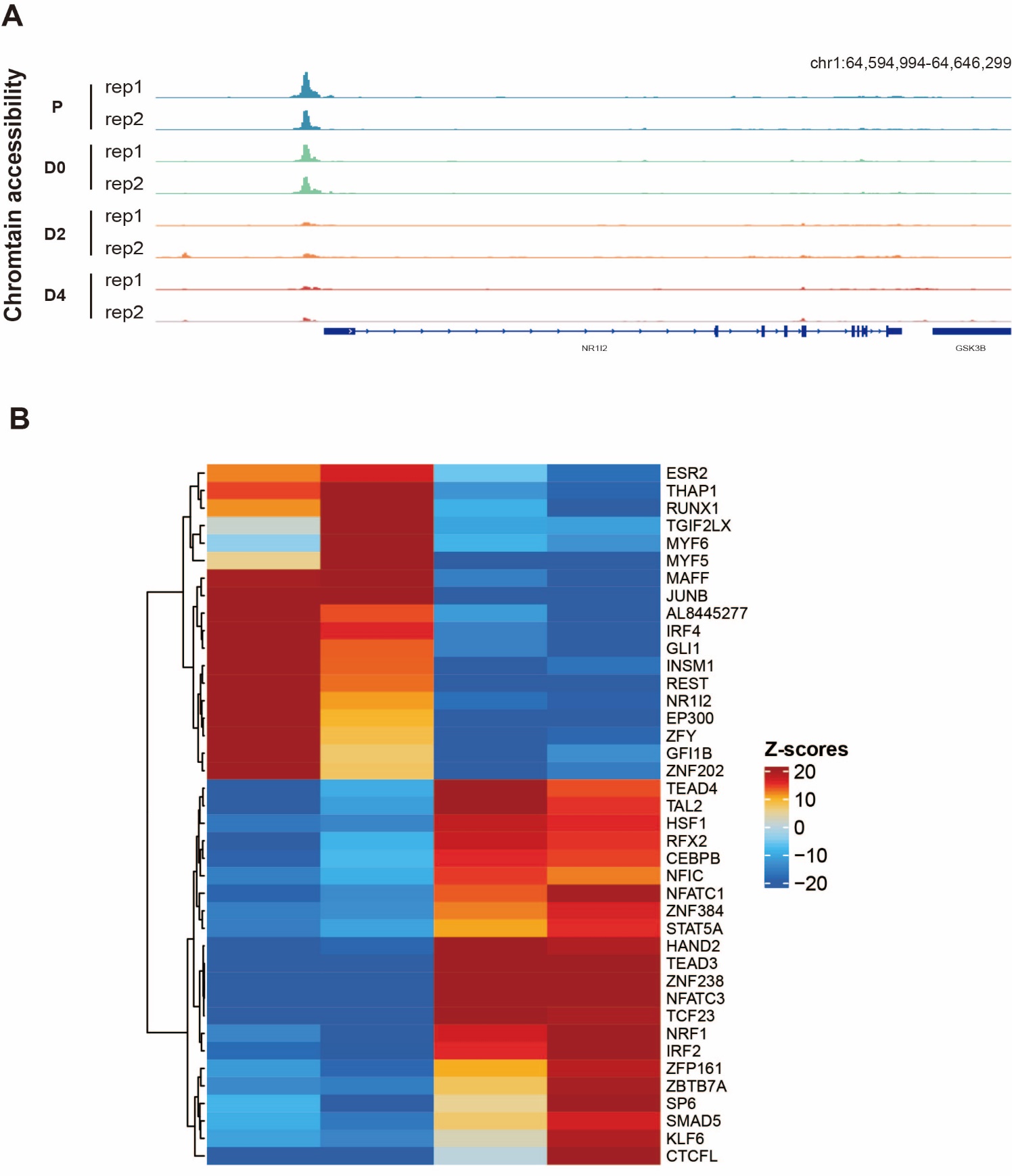


**Supplementary Figure 3: TF regulatory during bovine myoblasts proliferation and myogenic differentiation.** (A) Normalized coverage (CPM) of chromatin accessibility around *NR1I2* loci for each time point. (B) Heatmap displaying the top 40 most variably motifs.


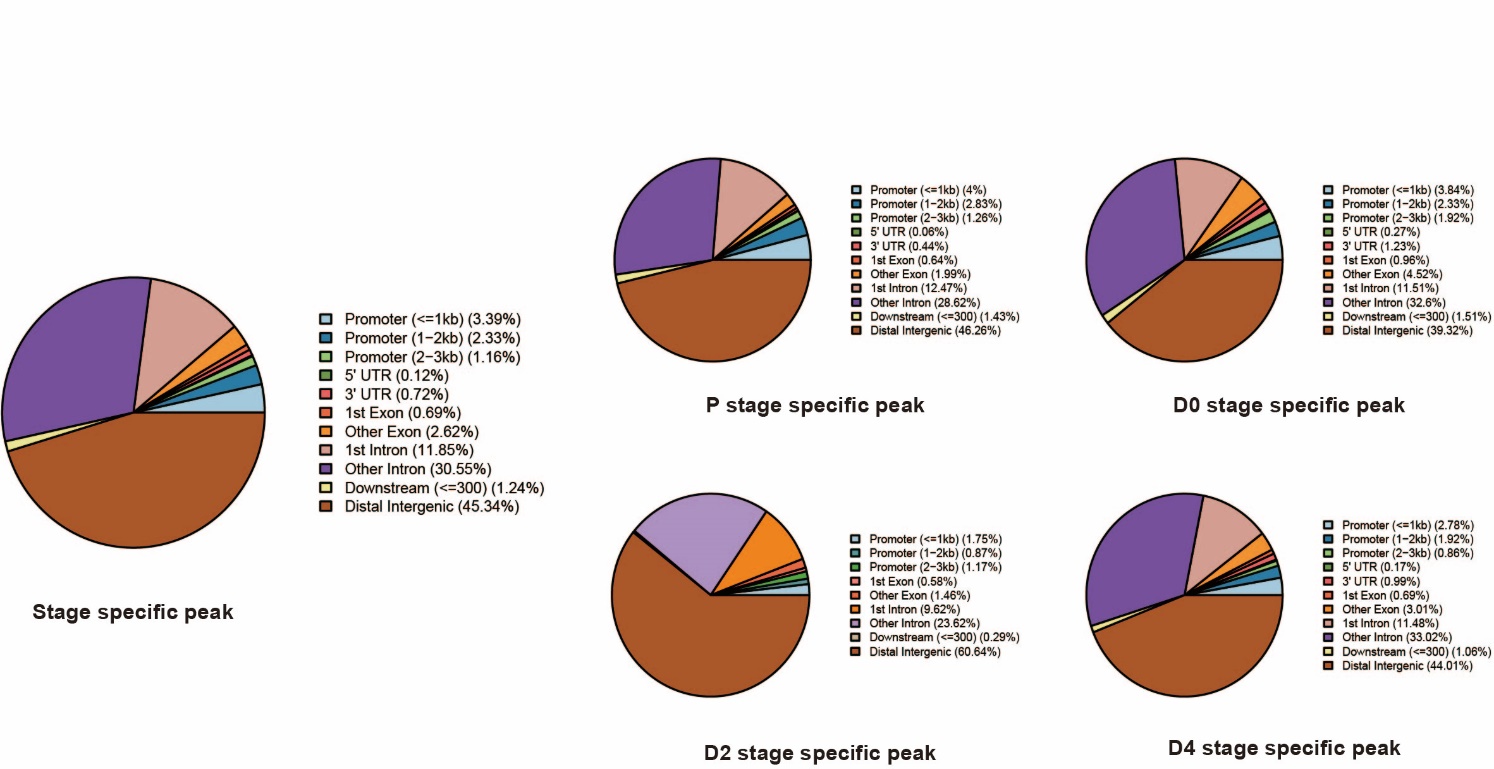


**Supplementary Figure 4:** Genomic distribution of stage-specific peaks at P, D0, D2 and D4 time point.


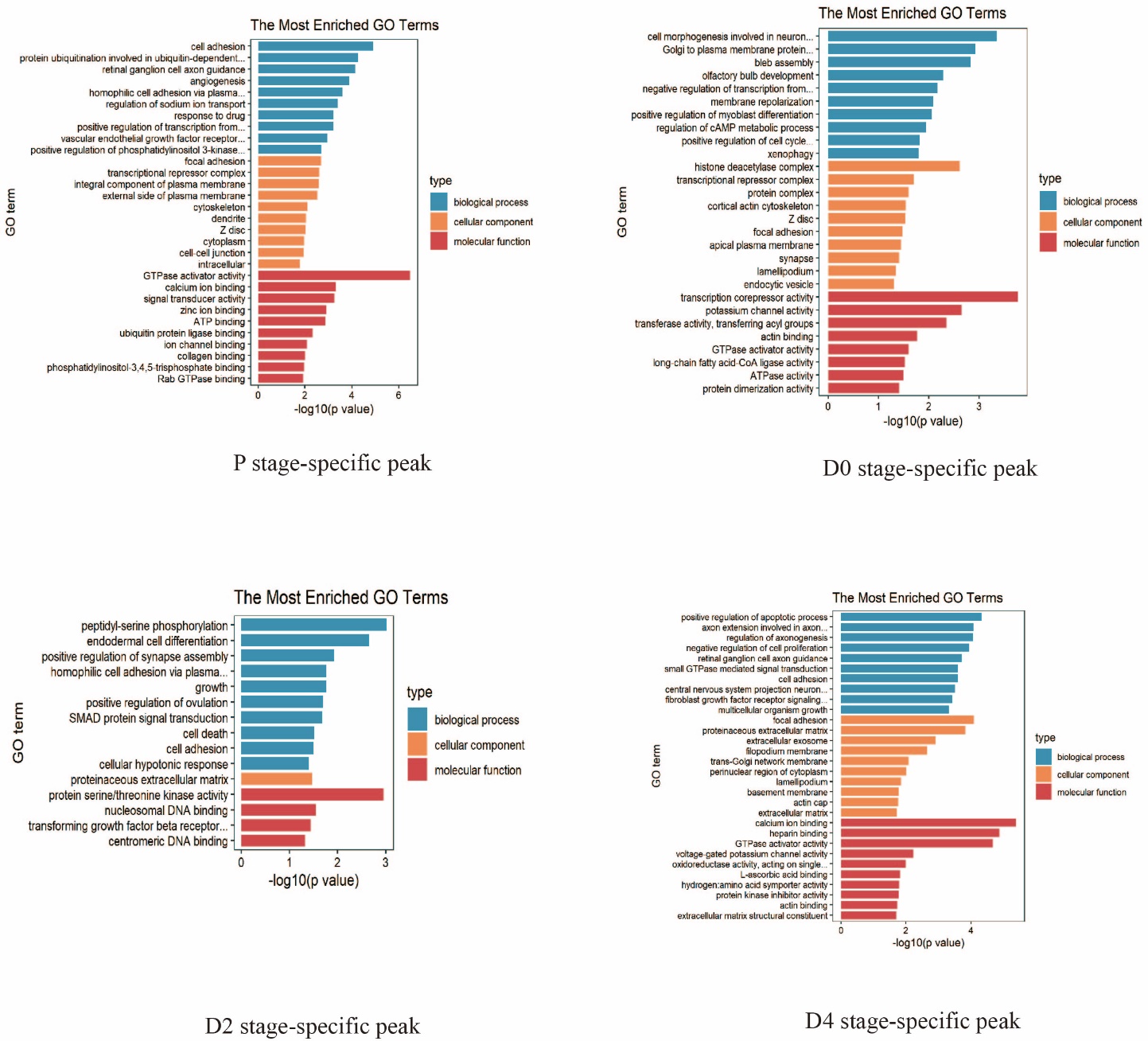


**Supplementary Figure 5: GO term biological function governed by stage-specific peaks.** Top GO terms (GOTERM_BP_DIRECT, GOTERM_CC_DIRECT and GOTERM_MF_DIRECT) for stage-specific peaks annotated to nearby genes at P, D0, D2 and D4 time point, respectively. p-value <0.05 is statistically significant.

**Supplementary Data1:** **The ranked list displaying TF motifs activity during bovine myoblasts proliferation and myogenic differentiation.**

**Supplementary Data2:** **Stage-specific peaks identified during bovine myoblasts proliferation and myogenic differentiation.**
